# “Starting from the Image”: A Tele-pathology Pre-graduate Course Aimed at Motivating Medical Students

**DOI:** 10.1007/s40670-023-01770-7

**Published:** 2023-03-17

**Authors:** Evgenia-Charikleia Lazari, Andreas C. Lazaris, Evangelia Manou, Georgios Agrogiannis, Constantinos Nastos, Emmanouil Pikoulis, Georgia-Eleni Thomopoulou

**Affiliations:** 1 First Department of Pathology, School of Medicine, The National and Kapodistrian University of Athens, 75 Mikras Asias St., Goudi, Athens, 115 27 Greece; 2 Third Surgical Department, School of Medicine, “Attikon” University Hospital, The National and Kapodistrian University of Athens, Athens, Greece; 3 Department of Cytology, School of Medicine, “Attikon” University Hospital, The National and Kapodistrian University of Athens, Athens, Greece

**Keywords:** Pathology, Case-based learning, Tele-education, Experiential learning, Empirical learning

## Abstract

In the tele-course entitled “Starting from the image”, medical students are confronted with practical tasks in relevant professional contexts. Initially, a macroscopic or microscopic image of a patient case is presented to learners who then receive relevant information on the patient’s history, clinical findings, and other laboratory tests. A pathologist actively discusses the pathological findings; then, a clinician explains their implications for the patient’s individualized treatment and prognosis. In this way, pathology’s interaction with other medical specialties is highlighted. Students declared that through these simulated professional practice experiences, they strengthened their decision-making skills. Educators should consider upgrading from information-based teaching to practice-focused instruction.

## Background: Reflections on Essential Medical Education

Learning concepts and theories exclusively in the abstract through study and reflection can be a problem in medical education. It is a fact that there is an intimate and necessary relation between the process of actual experience and the process of education [1]. In today’s world, we are awash in information from multitudes of print and electronic sources. The fundamental job of teaching no longer involves merely distributing facts but rather helping students learn how to use facts by developing their ability to think critically, solve problems, make informed judgments, and act rightly as professionals, for their own benefit and for the benefit of society.

Bridging basic sciences teaching and medical practice is a serious challenge in medical education. To many students, knowledge of histology, biochemistry, and molecular biology appears unrelated to their future professions. However, the teaching of medicine through the case study is increasingly gaining ground in contemporary medical school curricula [2].

The experiential learning process presents a stark contrast to the conventional, formal, academic learning process that involves acquiring information through the study of an academic subject without direct or indirect experience. While the dimensions of experiential learning are analysis, initiative, and immersion, the dimensions of academic learning are constructive learning and reproductive learning. Though both methods aim at instilling new knowledge in the learner, academic learning does so through more abstract techniques, whereas experiential learning attempts to actively involve the learner in concrete experience. Given the truly interactive and dynamic nature of experiential learning activities, there are innumerable ways any such activities can turn out, especially in vocational education contexts [3].

## Intervention/Tele-course Description

**Fig. 1:**
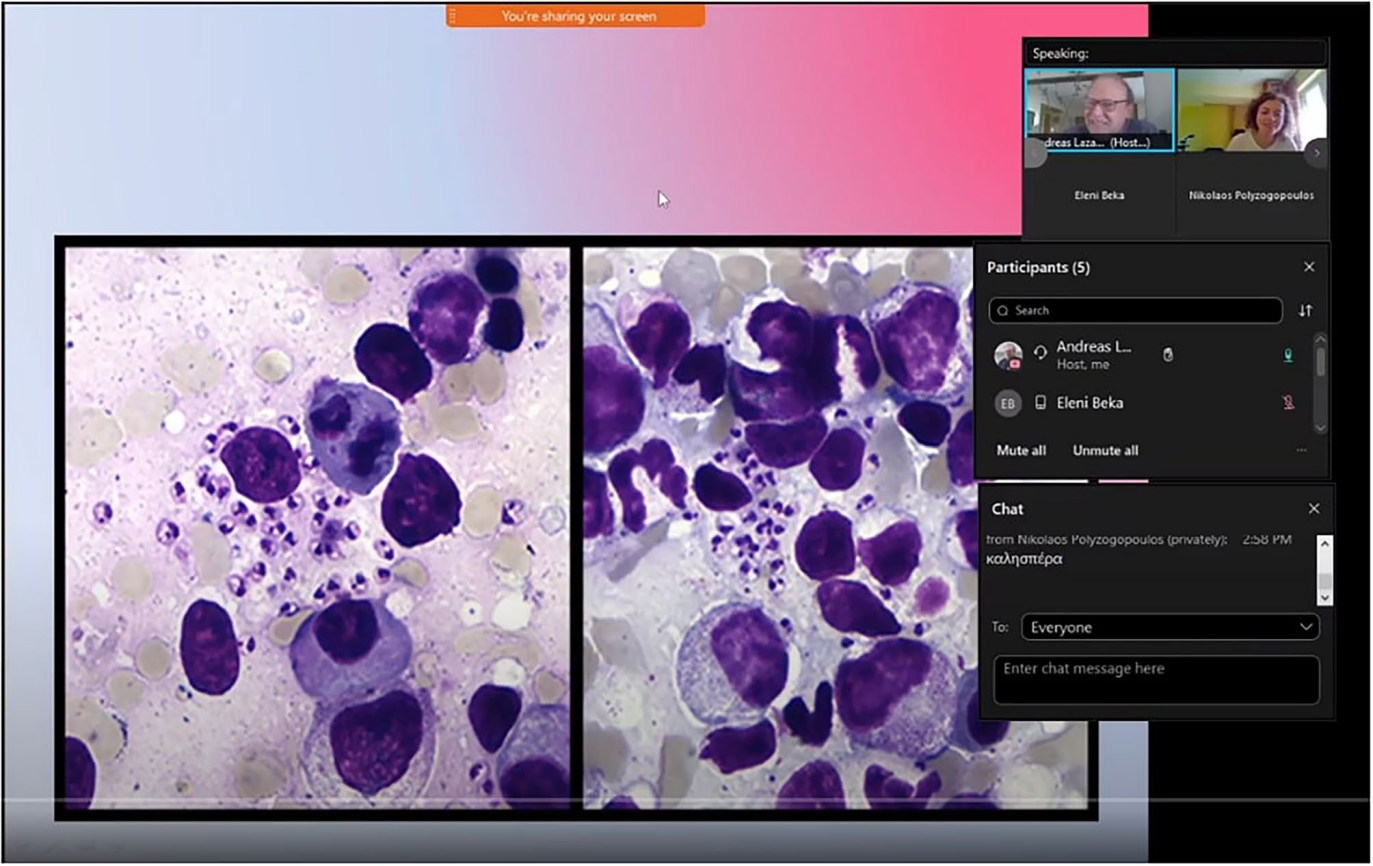
Snapshot from the tele-lesson of infectious diseases. A related question to the students could be: “What is the cause of pancytopenia in this case?” The patient’s bone marrow smear (May–Grunwald–Giemsa stain) shows numerous *Leishmania* amastigotes, with resulting pancytopenia

In the “Starting from the image” tele-course, which features optional synchronous attendance and/or asynchronous study, the teaching pathologist presents a macroscopic or microscopic pathological image from a carefully chosen patient case, as a starting point (Fig. 1). The pathologist then successively describes the features of the relevant disease as derived from the patient’s symptoms, the signs emerging from clinical examination, and the relevant laboratory findings, i.e., abnormal blood, urine values, etc., with comparative citation of the corresponding normal values. The educator often presents, in a parallel image, possible characteristic radiological imaging findings, as well. Next, the pathological findings from the initial pathological image that led to the final correct diagnosis are thoroughly analyzed and discussed interactively with the students in a question and answer session. At the end of each case, towards introducing students to clinical medical thinking, a guest clinician with a medical specialty relevant to the group of cases presented in each chapter/lesson, analyzes the clinical and laboratory examinations and explains the importance of the findings; the contribution of the pathology data and the relevant analysis to documenting the final diagnosis, the patient’s prognosis, and the therapeutic approach recommended is emphasized. The most individualized and patient-specific approach possible is applied in each case. Through this experiential learning principles–based approach, the learning activities in this tele-course engage students’ natural curiosity and are personally relevant to them as future professionals.

In order to achieve the participation of the tele-attendees, relevant prompts were often made clarifying that there was no requirement on the part of the educators for the students to have knowledge of clinical medicine in this phase of their studies (4th and 5th semesters); the listing of clinical data was intended to arouse their curiosity and try to understand how the laboratory findings they had been exposed to in the framework of the basic sciences, preclinical courses of the first five semesters of their studies (e.g., biology, biochemistry, pathophysiology) combine with the clinical manifestations of diseases.

This tele-course highlights, in a most tangible way, correct means of cooperating and displaying mutual respect among and between laboratory and clinical specialties, when caring for patients and handling their information. Thus, students pick up on the type of right collaboration between laboratory and clinical medicine that they should apply in the future as professional medical doctors. They begin to understand just how important biochemical-hematological and radiographic examination results are when combined with clinical and pathological data; accordingly, they begin to understand the pathologist’s need to stay informed regarding each patient’s relevant clinical history and lab work. For each pathology chapter, an appropriate clinician or clinicians participate, e.g., cardiologist and cardiac surgeon for the circulatory system chapter, gastroenterologist and gastrointestinal surgeon for the digestive tract chapter, nephrologist and urologist for the urinary tract chapter, and gynecologist and pediatrician for the female genital system and perinatal pathological anatomy chapter. It is worth noting that our invited fellow clinicians were clearly more willing to connect to the platform for simultaneous tele-courses than to present themselves for live teaching in the amphitheater.

Let us present two examples of case studies from the tele-course:

Ιn the gynecological pathology lesson, the teaching pathologist presents a microscopic image from a female with a normal endometrium at day 25 of the menstrual cycle in parallel with an image from an endometrial curetting from a teen girl with irregular menstrual cycles since menarche, two years prior, at day 25 of the menstrual cycle. The histologic characteristics of dysfunctional uterine bleeding due to anovulatory cycles in the abnormal image are analyzed in comparison with the normal image; the students are asked to identify small, proliferative-type glands in a hemorrhagic stroma. Then, pathophysiology is discussed (ovulation does not occur due to the absence of the luteinizing hormone surge; consequently, a corpus luteum does not form, and levels of progesterone do not increase to support the secretory phase; instead, proliferative glands that eventually undergo necrosis and bleed irregularly, persist in a stroma). Finally, a gynecologist explains hormonal treatment prescribed for such patients, often along with oral contraceptives containing progestational agents that promote controlled endometrial cycling.

Ιn the liver and biliary tract lesson, the teaching pathologist shows students a microscopic image of intrahepatic cholestasis, accompanied by the following data:

A middle-aged woman with a short history of scleral icterus and colicky right upper quadrant abdominal pain has only mildly increased aminotransferase levels, normal liver synthetic capacity with normal total serum protein and albumin, an alkaline phosphatase of 190 U/L [ normal limits (nl) 20–125 U/L], a total bilirubin of 10 mg/dL (nl ≤ 1.3 mg/dL) with a direct bilirubin of 9 mg/dL (nl ≤ 0.4 mg/dL). The microscopic findings are presented to the students with stress on the importance of the yellow–brown bile pigment accumulation which, in this case, is connected with elevated alkaline phosphatase levels. The biochemical role of bilirubin UDP-glucuronyl transferase, which is responsible for the conjugation of the material shown in the liver canaliculi in the microscopic image, is also highlighted. The clinician evaluates the significance of colicky right upper quadrant pain to the etiology of cholestasis (gallstone obstruction) and a general discussion of other etiologic factors for this pattern of cholestasis is held with the students.

## Impact: Evaluating the Importance of This Tele-course

The structure of the tele-course is akin to the general methodology of any scientific study that begins with observation and not with theory, whereas the traditional strategy of teaching medicine is, by that standard, rather improperly based [2].

In these case presentations, educators, as reflective practitioners, share their thoughts with the students to illuminate the process of clinical pathologic diagnosis-making. In this regard, the educators’ most important responsibility is to both seek out and construct meaningful educational activities that provide students with experience confronting real-world problems. The result is that knowledge—freed from the abstract inert forms that students memorize in dusty textbooks—comes alive in students’ understanding of its practical application. Experiential-type activities must allow the students themselves to make connections between the learning they are doing and the real world they live in. When one develops resonance with specific areas of knowledge through contact with such an educator, one really learns from that educator, who becomes a sort of mentor who models implementation of the knowledge, i.e., who shows one how to “live” the knowledge they are acquiring.

This tele-course was implemented, with optional attendance, in the 4th and 5th semesters of our medical students’ studies. The series of courses in question was included in the educational activity with optional participation of the students; we thought of making these courses available in the form of video files so that those students who for some reason did not attend them at the same time could have the opportunity to attend them at a later time. In the 4th semester, due to COVID-19 pandemic–related conditions, only distance education was implemented [4], during which the tele-lessons of this series related to the semester curriculum comprised the main educational activities on which the end of semester exams were based. In the 5th semester, conditions allowed for in-person teaching. Accordingly, the relevant tele-lessons were continued and completed, but only as a complement to the main education, and were thus only indirectly connected with the end of semester exams. All tele-lessons had been recorded and were soon available for study in students’ *e* portfolios [5].

During the 4th semester, in the midst of the quarantine due to the pandemic, about one-third of the semester’s students synchronously attended this optional tele-course, while more than two-thirds of the students were involved in short-term, asynchronous study of the relevant video files. During the 4th semester, the students attending the courses at the same time were numerous, so their interaction with the instructors was remarkable in the “chat” section of the platform, with the exchange of questions and answers on the findings of the images, but also on the clinical data and the relevant significance. During the 5th semester, when the restrictive measures were lifted due to the recession of the pandemic, synchronous attendance was minimized and this reduced attendance of tele-courses was also accompanied by reduced interactivity, while one-fourth of the class appeared to be involved in short-term asynchronous study. Students' preference for in-person classes with the educator’s physical presence is evident from the above and is certainly intertwined with their need for social contact, mainly with each other, especially after the exclusive distance learning conditions that were implemented for the full prior semester. Their need for socialization temporarily proved more urgent than their need for substantive education, given that during the in-person courses, educators generally focused more on traditional, theoretical, “academic” standards that are not so closely linked to clinical practice. However, οver time, the vast majority of students watched all the videotaped files (22 sessions garnered 13,112 views so far, among 200 students). The evaluation of the effort was done through an anonymous online questionnaire via Google Docs [6], where the students had the opportunity to freely express their criticism and suggestions. The vast majority of the medical students expressed great appreciation for the tele-course practical value and innovative character [6]. Ιn the students’ opinion, the tele-course most convincingly highlighted histopathology’s broad cognitive field and its valuable contribution to clinical medicine. Students who attended these courses synchronously or asynchronously felt that they were engaging in authentic situations and acquiring valuable understanding of clinical medicine before actually beginning their clinical practice the following semester (i.e., the 6^th^). This may save them some time in acquiring practical skills during their clinical practice. As one student stated, “learning is not just getting excellent grades on theory exams but gaining useful skills.” After the positive evaluation of our project, we really intend to include sessions held in interactive ways, in small groups, with active input from students, in our regular training program. We should consider that as the main obstacle to their participation in the discussion of the cases, the students mentioned their lack of familiarity with clinical medicine, which makes it preferable to transfer the tele-courses in question to a later semester.

Students hold positive opinions of this tele-course and are in agreement with participants in similar pathology tele-courses [7]. These tele-courses are part of an effort to upgrade medical education to a pre-eminently empirical context and to provide appropriate stimuli aimed at the essence of education rather than at the simple process of stirring and testing “dry” knowledge [8]. Undergraduate students must acquire holistic understanding of the science they are studying and then build their professional specialization on that solid foundation. The notion that “those who really want to learn will take the initiative and do so” is wrong in the extreme and is probably used as an alibi by indifferent teachers who do not want to make use of their experience and studies by sharing their knowledge with their students. An educational system must be designed to highly support all students, regardless of their individual self-efficacy levels, so that they will all be motivated by that educational system to some extent and become willing and capable of learning effectively. Despite the impressive technological advances in the teaching medium [9], the greatest and most pressing challenge ever faced by modern medical education, and education in general, is the restoration of its humanitarian character.

Synchronous tele-teaching, as well as teaching with the tutor’s physical presence—in contrast to the asynchronous study of recorded teaching sessions—allows for interactivity [10]. Given that, one may wonder about the institutionalization of interactivity in the educational strategy and practice and its multiple beneficial implications, one of the most important being an active attitude towards learning that will logically be transformed into a similar attitude towards the profession, in the future [11, 12]. Active learning methodologies are very effective for locating students’ creativity and talent [8]. Through their engagement in guided, authentic, “real world” experiences, they deepen their knowledge through repeated study of the selected cases and through reflection on the corresponding clinical decisions. They develop skills through practice and reflection, and they build potential support for the construction of new understandings, when placed in novel situations [13].

## Lessons Learned

Teaching and learning are undergoing modifications due to innovations in education. Teachers must understand the trends in contemporary teaching–learning processes and, in so doing, make learning more interesting, experiential, and interactive to motivate students not only to learn but to learn better, after coming into personal contact with a subject and its practical value.

## References

[1] Dewey J, Dewey J (1938). The philosophy of the arts. The Later Works.

[2] Lazaris AC, Lazaris AC (2018). Introduction: Implementing case-based learning in pathology. Clinical genitourinary pathology: a case-based learning approach.

[3] Riccioni O, Vrasidas C, Brcic L, Armenski G, Seiwerth S, Smeets A, van Krieken JH, Lazaris AC (2015). Acquiring experience in pathology predominantly from what you see, not from what you read: the HIPON e-learning platform. Adv Med Educ Pract.

[4] Government Gazette of the Hellenic Republic. April 9, 2020; 3707/B.

[5] Manou E, Lazari E-C, Thomopoulou G-E, Agrogiannis G, Kavantzas N, Lazaris AC (2022). Asynchronous e-learning after synchronous e-learning in the pathology course: when is the proper time for this transition?. J Educ Health Promot.

[6] Manou E, Lazari E-C, Lazaris AC, Agrogiannis G, Kavantzas NG, Thomopoulou G-E (2022). Evaluating e-learning in the pathology course during the COVID-19 pandemic. Adv Med Educ Pract.

[7] Sensu S, Teke O, Demirci M, Kutlu S, Genc BN, Ayalti S, Gurbuz YS, Erdogan N (2020). Telepathology in medical education: integration of digital microscopy in distance pathology education during Covid-19 pandemic. J Med Sci Clin Res..

[8] Rodríguez-Ardura I, Meseguer-Artola A (2016). E-learning continuance: the impact of interactivity and the mediating role of imagery, presence and flow. Inf Manag.

[9] Kay D, Pasarica M (2019). Using technology to increase student (and faculty satisfaction with) engagement in medical education. Adv Physiol Educ.

[10] Berge ZL (1999). Interaction in post-secondary web based learning. Educ Technol.

[11] Belezini E, Katsoulas N, Thomopoulou G-E, Lazaris AC (2020). The superiority of interactive courses combined with the teacher’s physical presence in the undergraduate pathology curriculum. J Contemp Med Educ.

[12] Manou E, Lazari E-C, Thomopoulou GE, Agrogiannis G, Kavantzas N, Lazaris AC (2021). Participation and interactivity in synchronous E-learning pathology course during the COVID-19 pandemic. Adv Med Educ Pract.

[13] Lazaris AC, Riccioni O, Solomou M, Nikolakopoulos I, Vemmou E, Karamaroudis S, Sotirianakou M-E, Vrasidas C, Armenski G, Seiwerth S, Smeets A, van Krieken JH, Patsouris ES. Implementation of experiential learning in pathology: impact of HIPON project concept and attainment. International Archives of Medicine Section: Proceedings of the 1^st^ International Online Medical Conference. 2015;8:211. ISSN: 1755-7682.

